# Live and Let Die: Roles of Autophagy in Cadmium Nephrotoxicity

**DOI:** 10.3390/toxics3020130

**Published:** 2015-04-13

**Authors:** Frank Thévenod, Wing-Kee Lee

**Affiliations:** 1 Institute of Physiology, Pathophysiology & Toxicology, Center for Biomedical Training and Research (ZBAF), Stockumer Str. 12, University of Witten/Herdecke, 58453 Witten, Germany; E-Mail: wing-kee.lee@uni-wh.de; 2 Laboratory of Signal Transduction, Sloan Kettering Institute, Memorial Sloan Kettering Cancer Center, 1275 York Ave., New York, NY 10021, USA

**Keywords:** unfolded protein response, transition metal, cadmium, apoptosis, autophagosome, acute kidney injury, malignant transformation, tunicamycin, rapamycin, mTORC

## Abstract

The transition metal ion cadmium (Cd^2+^) is a significant environmental contaminant. With a biological half-life of ~20 years, Cd^2+^ accumulates in the kidney cortex, where it particularly damages proximal tubule (PT) cells and can result in renal fibrosis, failure, or cancer. Because death represents a powerful means by which cells avoid malignant transformation, it is crucial to clearly identify and understand the pathways that determine cell fate in chronic Cd^2+^ nephrotoxicity. When cells are subjected to stress, they make a decision to adapt and survive, or—depending on the magnitude and duration of stress—to die by several modes of death (programmed cell death), including autophagic cell death (ACD). Autophagy is part of a larger system of intracellular protein degradation and represents the channel by which organelles and long-lived proteins are delivered to the lysosome for degradation. Basal autophagy levels in all eukaryotic cells serve as a dynamic physiological recycling system, but they can also be induced by intra- or extracellular stress and pathological processes, such as endoplasmic reticulum (ER) stress. In a context-dependent manner, autophagy can either be protective and hence contribute to survival, or promote death by non-apoptotic or apoptotic pathways. So far, the role of autophagy in Cd^2+^-induced nephrotoxicity has remained unsettled due to contradictory results. In this review, we critically survey the current literature on autophagy in Cd^2+^-induced nephrotoxicity in light of our own ongoing studies. Data obtained in kidney cells illustrate a dual and complex function of autophagy in a stimulus- and time-dependent manner that possibly reflects distinct outcomes *in vitro* and *in vivo*. A better understanding of the context-specific regulation of cell fate by autophagy may ultimately contribute to the development of preventive and novel therapeutic strategies for acute and chronic Cd^2+^ nephrotoxicity.

## 1. Introduction

Chronic Cd^2+^ toxicity due to chronic low Cd^2+^ exposure (CLCE) has emerged as a previously underestimated significant health hazard for human populations because it results from general dietary sources and cigarette smoking [1,2,3]. CLCE is a health problem for ~10% of the general population and increases morbidity and mortality [1]. With a biological half-life of ~10–30 years, Cd^2+^ accumulates in organs, particularly the kidney cortex [4], where it preferentially damages the proximal tubule (PT) cells (reviewed in [5,6,7,8]), which can result in fibrosis, failure [9], or—with Cd^2+^ being a class 1 human carcinogen—renal cancer [10]. Aside from nephrotoxicity, CLCE causes osteoporosis, neurotoxicity, genotoxicity, teratogenicity, and endocrine and reproductive defects [8].

## 2. Cell Death *versus* Survival and Malignancy

Organ failure and cancer are the two ends of a continuum of responses to Cd^2+^ toxicity. Cellular stress elicited by Cd^2+^ triggers detoxification as well as adaptive processes, which allow cell survival until normal function is restored. If the cellular response is insufficient, death pathways are initiated and the cell dies. However, if the level of stress is not sufficient to induce death but impacts on cells for a longer period of time (e.g., as in CLCE), cells may lose control over adaptive mechanisms, such as through alterations in signaling pathways induced by mutations, and malignant transformation can ensue [11]. Even if cells have been dysfunctional for a longer period of time and are therefore committed to die, survival may still persist through disruption of death signaling. This process facilitates malignant transformation (“evasion of apoptosis”) [12]. To avoid malignant transformation, cell death is imperative; therefore, it is crucial to clearly identify the death and survival pathways that determine cell fate in the setting of chronic Cd^2+^ nephrotoxicity.

## 3. Cellular Modes of Death: The Current Dogma

Current dogma states that cells die by several modes of death routines [13]: cells exposed to extreme physical or chemical stress die at once due to loss of their structural integrity, which is referred to as accidental cell death. In contrast, regulated cell death is initiated by genetically-encoded machinery and can therefore be manipulated by genetic or pharmacological tools. Within this type of cell death, programmed cell death (PCD) describes forms of cell death that occur as part of a developmental program or to preserve tissue and organ homeostasis during adulthood. Based on specific biochemical parameters and molecular events, PCD can be further classified into several major death modalities that include death receptor-dependent (extrinsic) apoptosis, caspase-dependent or -independent mitochondrial (intrinsic) apoptosis, regulated necrosis (necroptosis), mitotic catastrophe, and autophagic cell death (ACD) (caveat: see sections on autophagy) [14,15].

When cells are subjected to stress, cell death subtypes are triggered and a key feature of the death signal is the separation into an “initiation” phase when the process can still be reversed and cells have not yet committed to die, and an “execution” phase when a “point-of-no return” has been reached and cells are irreversibly sentenced to commit suicide. In the reversible initiation phase, cells respond by attempting to eliminate the source of stress and to repair damaged cellular structures and functions in order to re-establish their normal function [13]. If this adaptive response fails its aim, such as when the stress signal is prolonged or too severe for continuous and dominating survival mechanisms, execution of death occurs as an attempt to limit further damage, namely not to prolong malfunction and its consequences for the whole organism (e.g., cancer development). In this scenario, survival signals may either simply cease and be replaced by death promoting signals or they may co-exist for a certain period of time [13]. However, these definitions and classifications are an oversimplification and do not reflect the complexity of stress-induced cell fate in all instances.

## 4. Autophagy

### 4.1. Basic Principles and Regulation

Autophagy is a mechanism of “self-eating” (reviewed in [16,17]). By degrading intracellular components, damaged proteins, and organelles, the cell can increase its chances of survival during stress or nutrient starvation conditions; this works in a complementary fashion to ER-associated degradation (ERAD) [17]. Autophagy is part of a larger monitoring system for intracellular protein quality control and degradation that comprises the ubiquitin-proteasome pathway, a cytosolic protein degradation process for short-lived proteins, and the lysosomal system. Autophagy occurs at basal levels in all eukaryotic cells but can also be induced by intra- or extracellular cell stress signals [18]. Hence, it participates in many physiological processes within a cell and is considered to be an essential protein and metabolic homeostatic process. 

**Figure 1:**
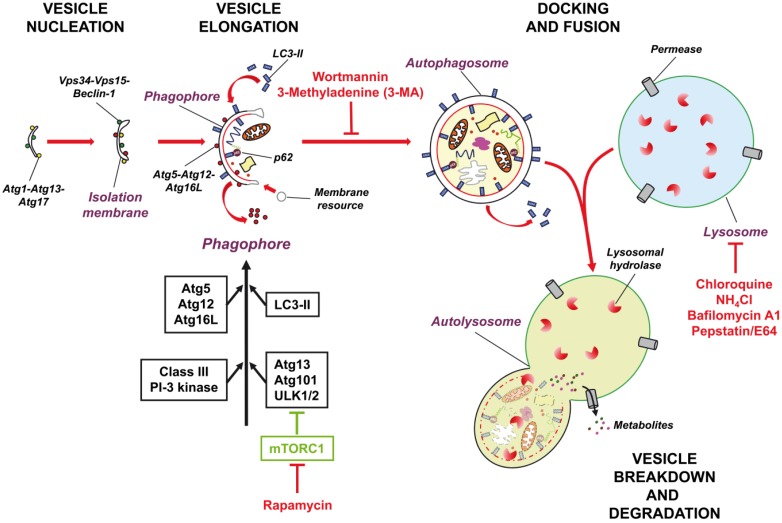
Model of autophagic flux and common inhibitors of the macroautophagic pathway. Macroautophagy involves autophagosome formation, delivery of autophagic components to the lysosome, and degradation of the sequestered cargo by lysosomal hydrolases. The “core” autophagic machinery utilizes Atg proteins for phagophore formation and its elongation to mature autophagosomes. The ULK1/2 kinase complex is required for induction of autophagy and is negatively regulated by mTORC1. A class-III phosphatidylinositol 3-kinase complex is required for nucleation of the phagophore membrane. Beclin 1 (Atg6) and Atg14 are part of this complex. The elongation and expansion steps in autophagosome formation involve two conjugation systems that require ubiquitin-like proteins, Atg12 and Atg8/LC3-I. The Atg5-Atg12 conjugate subsequently associates non-covalently with Atg16 to form an Atg12-Atg5-Atg16 multimeric complex. The second conjugation step is the formation of LC3-II (Atg8-PE) by conjugation of LC3-I with phosphatidylethanolamine (PE). Once autophagosomes are formed, most of the Atg proteins dissociate, which allows fusion with lysosomes to form autolysosomes. The sequestered contents and the inner membrane of autolysosomes are degraded by lysosomal hydrolases. Rapamycin inhibits mTORC and thereby promotes autophagy induction. The PI3K inhibitors wortmannin and 3-MA prevent the formation of autophagosomes. Blockers of lysosomal hydrolases operate either by neutralizing the intralysosomal acidic pH (the weak bases NH_4_Cl and chloroquine), inhibiting the lysosomal vacuolar-type H^+^-ATPase (bafilomycin A1), or preventing activity of lysosomal proteases (pepstatin/E64). Additional details are found in the text.

Cells display three types of autophagy [16,17]: microautophagy refers to the direct lysosome engulfment of a portion of cytoplasm by invagination, protrusion, or septation of the lysosome membrane. Chaperone-mediated autophagy denotes the process of direct transportation of unfolded proteins via the lysosomal chaperone protein hsc-70. These complexes then bind to LAMP-2A, a lysosomal membrane receptor, and are translocated across the lysosomal membrane. The third type of autophagy, macroautophagy, is by far the most important type. Macroautophagy refers to the *de novo* synthesis of a double membrane structure, the phagophore or isolation membrane, at the phagophore assembly site, which consists of the phagophore and the core molecular machinery (Autophagy-related genes, Atg) responsible for autophagosome formation. The phagophore expands and engulfs entire cytoplasmic components, including organelles, long-lived proteins, protein aggregates, and other cytosolic material. By fusing its two ends, the phagophore forms a double membrane compartment or vesicle, the autophagosome that “engulfs” cytosolic contents. The autophagosome matures by fusion with lysosomes to become an autophagolysosome, resulting in degradation of the engulfed content and inner membrane proteins by lysosomal acidic hydrolases (see Figure 1 for further details). The resulting amino acids are released into the cytosol and recycled. Autophagosome formation is controlled by a number of proteins and complexes including the mammalian target of rapamycin complex 1 (mTORC1), the serine/threonine-protein kinase ULK1/2, Beclin 1, autophagy (Atg) proteins, class III phosphatidylinositol 3-kinases (PI3K), and microtubule-associated protein 1A/1B-light chain 3-I/II (LC3-I/II) [18,19].

How is autophagy regulated? Generally, this pathway can be stimulated by multiple forms of cellular stress, for instance nutrient or growth factor deprivation, hypoxia, reactive oxygen species (ROS), damage of various cellular components, or intracellular pathogens [18]. Specific, stimulus-dependent and more general, stimulus-independent signaling pathways are activated to coordinate different phases of autophagy. Furthermore, autophagy can be integrated with other cellular stress signaling networks and has also multiple links to cell growth, proliferation, senescence, and apoptosis. In particular, upstream, autophagy can be regulated by ROS [20,21] and Ca^2+^ release [22]. ROS are generated by mitochondria [23] or possibly by NADPH oxidases in cancer cells [24] and appear to induce autophagy. By increasing autophagy, oxidative damaged proteins—which are thought to be delivered into autophagosomes by the autophagy cargo protein, p62—are degraded. The regulation of autophagy by ROS occurs at both the transcriptional and non-transcriptional levels. Important redox reactive transcription factors are HIF1, p53, Nrf2, and Foxo3. By modifying the expression or activity of autophagy-related proteins, autophagy or disinhibition of autophagy is increased. For example, Bcl-2 sequestration allows release of Beclin 1 to induce autophagosome formation [25,26]. Oxidative stress can also act directly on AMPK, leading to disinhibition of mTORC1 and increased autophagy [21]. [Ca^2+^]_cyt_ can activate as well as inhibit autophagy [22,27]. Inhibition of autophagy by [Ca^2+^]_cyt_ has been demonstrated through knockout or blockade of the IP_3_ receptor (IP_3_R), which increases autophagy. Because mitochondria require a constant source of Ca^2+^ for their metabolic needs that are provided by IP_3_R opening, loss of IP_3_R function results in decreased ATP production, AMPK activation and autophagy induction [27]. But Ca^2+^ may also activate autophagy. Some studies showed that autophagy induction is reduced with BAPTA-AM that chelates [Ca^2+^]_cyt_ [28,29,30,31].

### 4.2. Endoplasmic Reticulum Stress-Autophagy Crosstalk and Cell Fate: A Matter of Time

The endoplasmic reticulum (ER) stress pathway exemplifies the complex interactions of survival and death signals that determine the final outcome of cell stress. ER stress develops when ER function is perturbed by accumulation of misfolded proteins, depletion of Ca^2+^ stores, or oxidative stress in the ER lumen [32], and is sensed by three upstream signaling proteins: PERK/eIF2α, IRE1, and ATF6, collectively known as the unfolded protein response (UPR). They are found in the luminal ER membrane where, under ER stress, GRP78 chaperone binds to unfolded proteins, allowing the ER sensors to homodimerize and activate their downstream signaling cascades. Initially, the cells try to prevent accumulation of unfolded proteins by halting transcription through phosphorylation of PERK/eIF2α and to reduce the amount of unfolded proteins by initiating ERAD or autophagy to promote cell survival [33,34]. In the process of ERAD, which is regulated by XBP1 and ATF6, abnormally folded proteins are transported from the ER lumen to the cytoplasm, become ubiquitylated, and are degraded by the proteasome. However, chronic or excessive ER stress can lead to activation of apoptosis mediated by the induction of CHOP (a transcriptional repressor of anti-apoptotic Bcl-2 [35]), phosphorylation of JNK, or activation of caspase-12, which cleaves structural cytoskeletal proteins and activates DNase culminating in DNA condensation/fragmentation associated with apoptosis (reviewed in [33,36,37].

During Cd^2+^ damage of the kidney, ER stress occurs [38] and is initiated by alterations of ROS [39] or Ca^2+^ homeostasis [40], or both [41] through mechanisms that have been reviewed in detail elsewhere [7]. Other commonly used ER stress inducers are the inhibitor of *N*-linked protein glycosylation tunicamycin or the blocker of sarco-/endoplasmic Ca^2+^ pumps thapsigargin. The pattern of genes affected by Cd^2+^ appears to be distinct from other transition metals [42]. Cd^2+^ increases GRP78 (and possibly GRP94) in renal PT cells, indicating augmented unfolded proteins [41,43]. All three arms of the UPR, *i.e.*, anti-apoptotic PERK as well as pro-apoptotic ATF6 and IRE1, are activated by Cd^2+^ in kidney PT cells [39,41,43]. In our recently published work [41], we showed increased expression of a novel ER stress-induced survival factor, bestrophin-3, which prevents upregulation of pro-apoptotic CHOP [44]. Cd^2+^ induction of CHOP is associated with ATF6 in some studies with renal PT cells [39,43], but with the PERK arm in another study [45]. To cause apoptosis, Cd^2+^ activation of the IRE1 pathway may also stimulate phosphorylation of JNK [43,46], activate caspase 12 [40], or crosstalk with mitochondria to trigger intrinsic apoptosis signaling [47]. In summary, the UPR induced by Cd^2+^ can be perceived as an initial mechanism to alleviate ER stress but, more often than not, CHOP is upregulated and causes apoptosis even though counteracting survival mechanisms are intact.

Although UPR proteins may not necessarily be required for autophagy induction [30], numerous studies have provided evidence for autophagy activation as a direct consequence of ER stress (reviewed in [34]). Activation of the PERK/eIF2α–ATF4 pathway upregulates the expression of a large set of autophagy genes, such as Atgs, Beclin 1, or p62. While IRE1 signaling has been implicated in promoting autophagy via JNK-mediated signaling, it was also shown to elicit negative regulation of autophagy. Loss of autophagy genes induces the UPR, indicative of a negative feedback mechanism. Yet the definitive molecular bridges between the two processes remain to be elucidated. Functionally, autophagy promotes cell survival and increases energy supply. During ER stress conditions, autophagy is induced, possibly as a mechanism to eliminate aggregated proteins and damaged cellular components [33,34]. Hence, autophagy may decrease cellular stress levels by removal of ER membranes, which contain UPR sensors, or decrease the amplitude of stress by clearing aberrant proteins from the ER. In most cases, this autophagic induction is protective and may prevent ER-stress-associated cell death as part of an early adaptive and survival response [48]. However, in some instances, autophagy is invoked as a means of killing cells when ER stress is prolonged and substantial [49,50] (see also next paragraph). For instance, the study by Ding *et al.* [49] shows a differential role of autophagy in promoting survival of cancer cells or apoptosis of non-transformed cells, which may be related to the level at which ER stress can be compensated for. Overall, these observations indicate that autophagy can contribute to ER-stress-induced cell survival or death depending on the cellular context.

### 4.3. Autophagy: Janus Face of Survival and Death?

There is currently no consensus on the relationship between autophagy and cell death. The predominant views can be summarized as follows [51,52,53]: (1) Autophagy precedes apoptosis; (2) autophagy and apoptosis are mutually inhibitory: mild (early) stress initiates autophagy to limit apoptosis and strong (late) stress induces apoptosis that disrupts autophagy; and (3) ACD is rare and irrelevant for mammalian development or physiological cell death. Hence, most cases of ACD are not “death by autophagy”, but “death with autophagy” and rely on “phenomenological descriptions, nonspecific pharmacological inhibitors (e.g., 3-methyladenine and chloroquine) or incomplete Autophagy-related gene (*Atg*) depletion by siRNA” [51]. In fact, several effector proteins of autophagy acquire apoptosis-inducing properties following cleavage by calpains or caspase 3 [54,55,56,57], such as Beclin 1, Atg4D and Atg5, which is compatible with the notion that apoptosis terminates and inhibits autophagy [51]. However, these authors at least concede that there may be specific instances where ACD occurs [51], e.g., a cell death form that was triggered by a cell-penetrating autophagy-inducing peptide, Tat-Beclin 1, derived from the autophagy protein Beclin 1 but relied on the plasma membrane Na^+^/K^+^-ATPase, was dubbed “autosis” [58]. 

Others have made the effort to identify more scenarios where (possibly non-apoptotic) autophagy-dependent cell death may occur in order to adequately describe certain death phenomena (reviewed in [59,60,61]) and in particular to understand the cellular context dependence of autophagic function and/or the role of autophagy during pathological stress [62,63,64,65,66]. 

Hence, autophagy can contribute to cell death under certain experimental conditions characterized by the absence of intact apoptosis pathways. Ullman *et al.* [50] demonstrated that autophagy has opposite effects on cell fate in response to ER stress (thapsigargin, tunicamycin, or brefeldin A) in apoptosis-competent cells in which autophagy increased survival mechanism, and in apoptosis-deficient cells that utilized autophagy as a means to promote non-apoptotic cell death via necrosis. Similarly, Shimizu and coworkers found that Jun N-terminal kinase (JNK) was activated in etoposide- and staurosporine-treated, but not serum-starved, Bax^−/−^/Bak^−/−^ cells, and that ACD was suppressed by disruption of JNK activity [67,68]. Other examples of non-apoptotic ACD include caspase inhibition in mouse fibroblasts [69,70], expression of a Beclin 1 mutant that escapes Bcl-2 regulation in breast cancer cells that have inactive caspase 3 [71], and caspase 10 inhibition in myeloma [72]. 

In apoptosis-competent cells, autophagy can also lead to autophagy-dependent but caspase-independent cell death, e.g., in cells expressing a short isoform of p19ARF [73], or in human ovarian epithelial cells expressing oncogenic H-Ras^V12^, was associated with Bcl-2 family member Noxa displacement of Mcl-1 from Beclin 1, which led to autophagy-dependent cell death [74]. Still other studies have shown that the tumor suppressor DAPk (calmodulin-regulated serine/threonine kinase Death-Associated Protein kinase or Death-Associated Protein kinase 1 according to certain nomenclatures) mediates apoptosis induced by several external stresses, including ceramide [75,76]. DAPk is an activator of autophagy and apoptosis and has also been proposed to convert autophagy from a cell survival mechanism to a cell death initiating mechanism [77]. A current interpretation of the dual impact of this kinase on autophagy and apoptosis would be that cytoprotective autophagy is triggered by low level kinase-regulated stress, whereas more intense and protracted stress culminates in apoptosis [51]. DAPk is highly expressed in the kidney, especially in proximal tubule (PT) cells [78], and deletion of the kinase domain of DAPk in mice attenuates tubular caspase-dependent cell apoptosis in renal ischemia-reperfusion injury [79]. Injection of tunicamycin, an ER stress inducer, into kidneys of mice causes kidney PT autophagy and caspase-dependent apoptosis in the same cells, and apoptosis is attenuated in DAPk^−/−^ mice (autophagy was not studied in DAPK^−/−^ mice, but was also reduced in DAPK^−/−^ cells derived from these mice) [80]. In this context, a relevant and intriguing observation is the “ceramide autophagy paradox” [62,81]: stress induced by ceramides of specific chain length can lead to cytoprotective autophagy and survival or promote death by activating pro-apoptotic signaling and/or binding to membranes of damaged mitochondria, where they act as receptors for phosphatidylethanolamine (PE)-conjugated LC3-II and thereby target phagophores for lethal mitophagy induction [82]. Finally, a recent study also shows that DNA damaging agents trigger Atg5 upregulation concomitantly with autophagy induction, and Atg5, in addition to promoting autophagy, translocates to the nucleus to elicit G2/M arrest and mitotic catastrophe [83], another form of cell death caused by DNA damage [84]. Collectively, these studies indicate that autophagy and apoptosis may not be mutually exclusive events, but rather can occur within the same cell in a linked fashion following stress induction. Hence, autophagy and its effectors very likely play a dual role in determining cell fate, depending on specific cell types and stress stimuli.

### 4.4. Autophagic Cell Death and Cd^2+^ Toxicity

Apart from the kidney, in most non-cancerous cellular and animal models, autophagy mediates or promotes cell death induced by Cd^2+^. In Cd^2+^-exposed sea urchin embryos, inhibition of autophagy produced a concurrent reduction of apoptosis, suggesting that the two phenomena are functionally related [85]. Apoptosis, assessed by TUNEL assay and cleaved caspase-3, was restored in Cd^2+^-treated embryos when autophagy was inhibited by the inhibitor of autophagosome formation, 3-methyladenine (3-MA). In normal human liver L02 cells, Cd^2+^ toxicity correlated with increased LC3-II formation, GFP-LC3 puncta, mitochondrial fragmentation as a result of increased dynamin 1-like (DNM1L) expression and translocation to mitochondria, increased mitochondrial co-localization with lysosomes as well as mitochondrial loss and bioenergetic deficit. ATG5 siRNA suppressed mitochondrial loss and cytotoxicity, indicating that augmented mitophagy in Cd^2+^-exposed cells increases cell death [86]. Furthermore, a DNM1L inhibitor blocked mitophagy and ameliorated Cd^2+^-induced hepatotoxicity *in vivo*, suggesting that Cd^2+^-induced mitophagy contributes to hepatotoxicity. In addition, in non-transformed human lung bronchial epithelial BEAS-2B cells, Cd^2+^ exposure induced apoptosis that was associated with increased formation of ROS, LC3-II and the frequency of GFP-LC3 puncta whereas these changes were not observed in Cd^2+^ apoptosis-resistant cells, implying that transformed cells are autophagy deficient [87].

### 4.5. Autophagy in Acute Kidney Injury and Cd^2+^ Nephrotoxicity: A Reflection of the General Controversy in the Field

The role of autophagy in kidney damage reflects the general controversy in the field of cell death signaling [88,89,90,91]. The dispute is further exacerbated because of the current relevance of acute kidney injury (AKI) for intensive care and transplantation medicine in an ageing population [92] and the hope for the rapid discovery of efficient therapies. Hence, conflicting results can be found in AKI induced by stress stimuli (reviewed in [89]). In tunicamycin-treated mice, ER stress induces apoptosis and autophagy concomitantly in the same damaged tubular cells and DAPk^−/−^ mice are resistant to ER stress-induced kidney injury as tubular cell autophagy is suppressed [80]. In contrast, using the gold standard for identifying the function of autophagy in cell stress, namely the generation of tubule-specific autophagy (Atg5^−/−^) knockout animals, evidence for a protective role for autophagy in ischemia-reperfusion AKI has been obtained. During renal ischemia-reperfusion, tubular cell autophagy is inhibited in Atg5 conditional knockout mice and, importantly, more severe kidney injury is induced by renal ischemia-reperfusion in these mice compared with wild-type animals [93,94]. 

For cultured cell lines and experimental animals exposed to Cd^2+^, only a handful of reports have been published so far, with partly conflicting data. Shih *et al.* showed in cultured mesangial cells that Cd^2+^ (6 µM for 28 h) triggers autophagy and caspase-dependent apoptosis [95]. 3-MA reduced autophagy without affecting apoptosis and the pan-caspase inhibitor Z-VAD-fmk attenuated apoptosis without affecting autophagy, indicating activation of independent signaling pathways; in contrast, both inhibitors increased cell viability. This suggested that both ACD and caspase-dependent mechanisms contribute to cell death and coincide in mesangial cells exposed to Cd^2+^. Chargui *et al.* [96] treated rats with low (“subtoxic”) Cd^2+^ concentrations (0.3 mg/kg Cd^2+^ i.p. for up to 5 days) or exposed cultured PT cells to 5 µM Cd^2+^ for 5 h. Cd^2+^ *in vivo* increased PT autophagy and increased reactive proliferation with no evidence of glomerular and tubular dysfunction or caspase-dependent apoptosis despite massive ultrastructural signs of cellular degeneration. In cultured immortalized mouse PT cells, Cd^2+^ increased ER stress, autophagy, and cell death, which were prevented by 3-MA, but not by the V-ATPase blocker bafilomycin A1, which blocks acidification of late endosomes and lysosomes. Hence, the data suggested that ER stress and autophagy are interlinked events for disposal of aggregated ubiquitinated proteins in Cd^2+^-exposed PT cells but they may also contribute to induction of a caspase-independent form of cell death (ACD?) *in vivo* and *in vitro*. Furthermore, mice treated with Cd^2+^ (0.2–0.8 mg/kg Cd^2+^ i.p. for 3 days) showed disruption of kidney mitochondrial membrane potential (Δψ_m_), increased LC3-II/LC3-I ratio, mitophagosome formation, and decreased mitochondrial mass, suggesting Cd^2+^-induced mitophagy [97]. Additional experiments led to the conclusion that Cd^2+^ elicits ROS-mediated mitophagy through a PTEN-induced putative kinase 1 (PINK1)/Parkin pathway in kidneys of mice, thus promoting Cd^2+^ nephrotoxicity. Finally, Matsuoka *et al.* [98] demonstrated that Cd^2+^ (20 µM for 4 h) activates an Akt-, mTORC1-, and anti-apoptotic UPR- (ATF4) dependent survival pathway in human HK-2 PT cells that is blocked by the mTORC1 inhibitor rapamycin (also named sirolimus) (*ca.* 200 nM for 1 h), thus suggesting that rapamycin (and autophagy induction) promotes Cd^2+^-induced death of PT cells. 

In contrast, Kato *et al.* [46] exposed NRK-52E rat PT cells to 10 µM Cd^2+^ for 72 h or administrated mice twice with Cd^2+^ (10 mg/kg, i.p.) at 12 h intervals and removed the kidney 3 days later. Cd^2+^ increased cell death and caspase-dependent apoptosis through a ROS, mTORC1, pro-apoptotic UPR (IRE1)- and JNK-dependent pathway both *in vivo* and in cultured cells. Cell death induced by Cd^2+^ *in vivo* and *in vitro* was abolished by co-exposure with rapamycin (100 nM), an immunosuppressant with low renal toxicity, which inhibits mTORC1 and strongly induces autophagy by blocking the ULK1/2 complex [99], suggesting that autophagy induction is protective.

### 4.6. Is Autophagy Protective Against Cd^2+^-Induced Nephrotoxicity? A Critical Appraisal

With one notable exception [46], most data obtained in kidney cells suggest that, in contrast to ischemia/reperfusion injury, Cd^2+^ induces ER stress to promote caspase-dependent and/or -independent cell death, which may be mediated by ACD. This conclusion is supported by our recent data (see [100] and below), which could not reproduce the protective effects of rapamycin on Cd^2+^-induced death in NRK-52E cells [46].

Our laboratory has intensively investigated the mechanisms of Cd^2+^-induced cell death and survival of PT cells in culture (reviewed in [5,6,7,8]). We have established Ca^2+^ and ROS as triggers of ER stress/UPR-induced early cell death, mediated by Ca^2+^-dependent calpain activation. Cd^2+^ (25 µM) elicits Ca^2+^- and ROS-dependent ER stress and death, but also rapidly (3–6 h) triggers an adaptive UPR via ERK1/2-dependent bestrophin-3 upregulation, which enhances survival by inhibiting CHOP-dependent apoptotic cell death [41]. Cd^2+^ (5–50 µM) also causes early (3–6 h) *de novo* ceramide formation, which increases [Ca^2+^]_cyt_, resulting in Ca^2+^-dependent calpain activation and apoptosis followed by late apoptosis (24 h) via calpain-dependent caspase activation [101,102]. Mitochondrial caspase- and AIF-dependent apoptosis are also mediated by direct effects of Cd^2+^ on mitochondria: Cd^2+^(5–50 µM) induces mitochondrial damage by entering the mitochondrial matrix through the mitochondrial Ca^2+^ uniporter, subsequent disruption of Δψ_m_, activation of mitochondrial aquaporin 8, osmotic swelling of the matrix, and mitochondrial rupture—which would entail induction of mitophagy—and results in the release of cytochrome *c* and AIF, caspase activation, and apoptosis [101,103].

**Figure 2:**
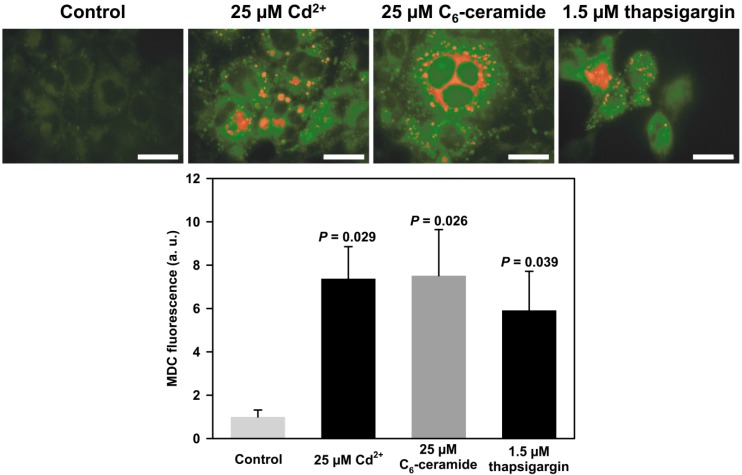
Autophagosome formation induced by Cd^2+^, C6-ceramide, or thapsigargin in PT cells. WKPT-0293 Cl.2 cells were incubated for 1 h with Cd^2+^, C6-ceramide, or the ER-stress inducer thapsigargin prior to monodansylcadaverine (MDC; 10 µM) fluorescence staining (upper panels). During the experimental period cells were incubated in culture medium with 1% FBS, as described in [46]. An average of 3–6 experiments, 7–15 images each, was performed for statistical analysis (lower panel) by one way ANOVA Dunnett post-hoc test. Scale bar = 100 µm for the control and 50 µm for the experimental conditions.

**Figure 3:**
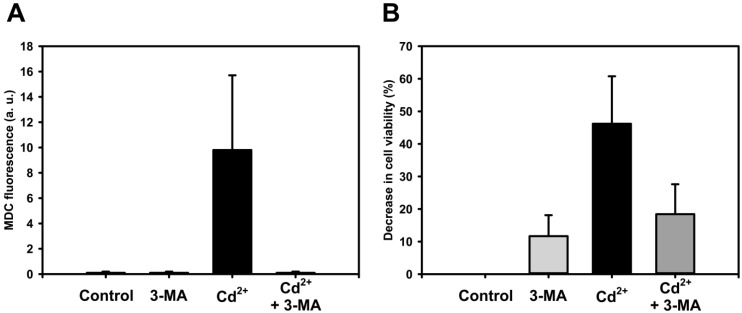
Effect of autophagy inhibition by 3-MA on Cd^2+^-induced autophagosome formation and cell viability in renal PT cells. WKPT-0293 Cl.2 cells were incubated for 1 h with 10 µM (**A**) or for 24 h with 25 µM Cd^2+^ (**B**) ± the autophagy inhibitor 3-methyladenine (3-MA; 5 mM), prior to monodansylcadaverine (MDC; 50 µM) fluorescence staining or cell death assay. An average of 10 images from one experiment is shown in (**A**). For measurements of cell viability by MTT assay, 3-MA was pre-incubated for 30 min prior to washout and incubation ± Cd^2+^ for 24 h. Means ± S.D. of three different experiments are shown in (**B**). During the experimental period, cells were incubated in medium containing 1% FBS, similarly as described in [46].

Already in 1999 we showed that Cd^2+^ (5–10 µM for up to 20 h) induces ROS formation and apoptosis, which is associated with proteolytic degradation of Na^+^/K^+^-ATPase via proteasomal and endosomal/lysosomal pathways [104]. This proteolytic lysosomal degradation likely reflects autophagy. Interestingly, GSK3-β, which controls both proteasomal protein degradation [105] and autophagy [106], is activated by Cd^2+^ in renal cells [107]. In preliminary experiments, we have tested the effect of Cd^2+^ on autophagy and cell fate in p53-deficient WKPT-0293 Cl.2 and p53-proficient NRK-52E rat PT cells and investigated the effect of 3-MA and rapamycin on these end-points. Both cell lines display similar results with the exception that NRK-52E cells are more sensitive to Cd^2+^ exposure (*EC_50_* for WKPT-0293 Cl.2 cell death ~18 µM and for NRK-52E cells ~4 µM at 24 h). In WKPT-0293 Cl.2 cells, a marker for autophagolysosomes, monodansylcadaverine (MDC; 10 µM), accumulates as early as 1 h after application of Cd^2+^ (25 µM), short chain C6-ceramide (25 µM), or the ER-stress inducer thapsigargin (1.5 µM) (Figure 2), which is in line with observations *in vivo* and *in vitro* that autophagy is an early event induced by Cd^2+^ and other ER stress inducers in renal PT cells [80,96]. The data also support the concept that Cd^2+^-induced ceramide formation and ceramide-dependent calpain activation [108] modulates autophagy (and possibly mitophagy) to promote cell death [62,81,82]. Autophagosome formation in WKPT-0293 Cl.2 cells induced by 10 µM Cd^2+^ for 1 h is abolished by 5 mM 3-MA, which also reduces the decrease of cell viability caused by Cd^2+^ (Figure 3). This indicates that autophagy contributes to cell death in renal PT cells exposed to Cd^2+^.

**Figure 4:**
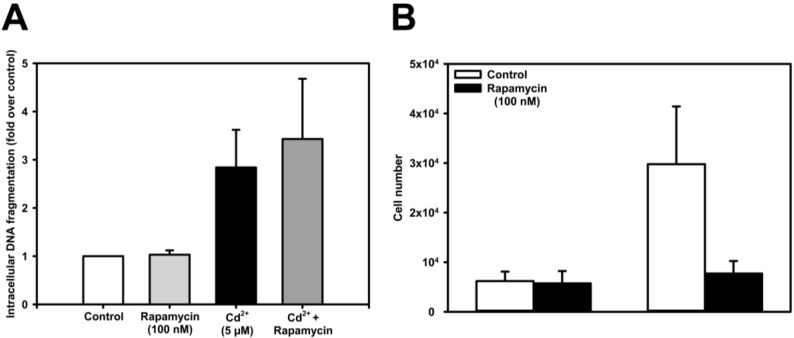
Effect of rapamycin on apoptosis and cell proliferation in Cd^2+^-exposed renal PT cells. NRK-52E cells were exposed to 100 nM rapamycin for 24 h in the absence or presence of 5 µM Cd^2+^ and apoptosis was assayed by measuring intracellular DNA fragmentation (**A**). Cell number of subconfluent cells was determined in culture dishes ± rapamycin at time 0 and after 24 h by trypsinization and cell counting with a hemocytometer (**B**). During the experimental phase, cells were cultured in medium containing 1% FBS, similarly as described in [46]. Means ± SE of 3–8 different experiments are shown.

In contrast to the studies by Kato *et al.* [46] that were performed in NRK-52E renal cell cultures and in experimental animals, we could not confirm that the mTOR blocker rapamycin protects renal PT cells (WKPT-0293 Cl.2 and NRK-52E) against Cd^2+^-induced cell death (Figure 4). By measuring DNA fragmentation as an end-point of apoptosis, we could not detect any protective effect of rapamycin on Cd^2+^-induced DNA fragmentation (Figure 4A). It is interesting to note that rapamycin *per se*, when used at the same low concentration (100 nM) as in the aforementioned study, decreases apparent cell viability, as measured by MTT assay. However, the MTT assay does not discriminate between cell viability and cell proliferation, and because mTORC1 is also known to increase growth and proliferation [109], we investigated this aspect further and could confirm that cell number, and hence proliferation, is drastically reduced by rapamycin (Figure 4B).

To account for their observations, Kato *et al.* [46] accordingly proposed a model in which inhibition of mTORC1 by rapamycin prevents activation of pro-apoptotic UPR signaling. However, renal PT cells also express mTORC2, which has been shown to activate Akt/PKB signaling and to inhibit downstream pro-apoptotic UPR signaling, thus inducing protection against cisplatin nephrotoxicity in experimental animals and NRK-52E cells [110]. Furthermore, it has been known for some time that although short exposure with rapamycin selectively inhibits mTORC1, prolonged exposure (≥24 h) also inhibits mTORC2 assembly and Akt/PKB [111]. Considering that Kato *et al.* [46] exposed NRK-52E cells for 72 h to prevent Cd^2+^ toxicity, their results are surprising and difficult to reconcile with our data (Figure 4A and [100]). They are also not supported by clinical studies in kidney transplant patients demonstrating that rapamycin therapy exerts increased toxicity on tubular epithelial cells and/or retards healing, leading to an increased incidence of delayed graft function [112].

**Figure 5:**
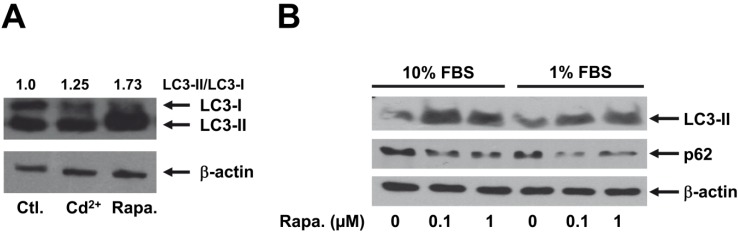
Effect of Cd^2+^ and rapamycin on autophagy markers LC3-I/II and p62 in renal PT cells. (**A**) WKPT-0293 Cl.2 cells were exposed to 10 µM Cd^2+^ for 1 h or to 100 nM rapamycin for 24 h and LC3 immunoblots were performed; (**B**) NRK-52E cells were cultured in medium containing 1% or 10% FBS, exposed to different concentrations of rapamycin for 24 h, and autophagy markers LC3 and p62 were determined by immunoblotting. Loading was monitored with β-actin. Data are representative of 2–4 experiments.

Microtubule-associated protein light chain 3 (LC3) is now widely used to monitor autophagy [113]. One approach is to detect LC3 conversion (LC3-I to LC3-II) by immunoblot analysis because the amount of LC3-II is clearly correlated with the number of autophagosomes. Another marker is p62, a receptor for cargo (e.g., ubiquitinated protein aggregates) destined for autophagic degradation, that is proteolysed as well during autophagic activity [113]. Both Cd^2+^ (10 µM; 1 h) and rapamycin (100 nM; 24 h) cause an increase in LC3-II level in renal WKPT-0293 Cl.2 PT cells (Figure 5A), and rapamycin increases p62 degradation in NRK-52E cells (Figure 5B). Rapamycin clearly increases autophagy in renal PT cells (Figure 5), but because rapamycin does not reduce Cd^2+^-induced apoptosis of renal PT cells (Figure 3), a protective role of autophagy in Cd^2+^-induced renal cell death, as proposed by others [46], is unlikely. Interestingly, p62 expression with Cd^2+^ is biphasic in PT cells with a decrease of p62 at 0.5–1.5 h and its accumulation at 6–8 h (data not shown), suggesting a time-dependent dual effect of Cd^2+^ on autophagy induction and/or lysosomal activity. Hence, whereas Cd^2+^ may elicit early ROS and/or Ca^2+^ signals to activate protective ER stress and autophagic flux, prolonged Cd^2+^ exposure could disrupt autophagy and provoke ACD, possibly by inducing lysosome permeability and/or damage [114].

The two *in vivo* studies that have investigated the impact of Cd^2+^ on renal integrity and autophagy are more difficult to evaluate. Chargui *et al.* [96] exposed rats to low Cd^2+^ concentrations (0.3 mg/kg Cd^2+^ i.p. for up to 5 days) and characterized morphological and biochemical signs of damage, death, and autophagy that appeared to occur concomitantly in renal PT cells within 1 day of Cd^2+^ exposure. Renal morphology showed signs of recovery after 5 days’ Cd^2+^ exposure while autophagy was still prevalent. Interestingly, no increased apoptosis (caspase-3 positive cells) was detected at any time. whereas cellular proliferation (as measured by Ki67 immunohistochemistry) was significantly increased throughout the treatment period. Kato *et al.* [46] treated mice twice with Cd^2+^ (10 mg/kg, i.p.) at 12 h intervals and removed the kidney three days later. The role of the autophagy inducer rapamycin on renal damage was investigated by injecting rapamycin (1.5 mg/kg i.p.) on day 1, 2, 3, and 4. Cd^2+^ treatment induced massive damage of the renal cortex, which was largely prevented by co-application of rapamycin. However, Kato *et al.* [46] did not look for markers of autophagy but rather focused on the PERK-eIF2α pathway of the UPR. Interestingly, whereas Chargui *et al.* [96] found no increase of caspase 3-positive cells in the rat kidney cortex with Cd^2+^ treatment (despite dramatic renal cellular damage), mice exposed to Cd^2+^ in the study by Kato *et al.* [46] developed massive apoptosis (as determined by TUNEL-positive cells). This indicates that Kato *et al.* [46] induced AKI, whereas the weak Cd^2+^ stress in the study of Chargui *et al.* [96] induced a non-apoptotic mode of PT cell damage. Nevertheless, whereas the *in vivo* data by Kato *et al.* [46] suggest that rapamycin (and possibly autophagy induction) is protective against strong renal Cd^2+^ stress, no such conclusion on the role of autophagy in Cd^2+^ nephrotoxicity can be drawn from the *in vivo* data of Chargui *et al.* [96] since both non-apoptotic PT damage and autophagy concurred during weak Cd^2+^ stress. 

## 5. Conclusions and Future Perspectives

Based on the current state of knowledge and the literature, it appears mandatory to investigate the relationship between autophagy and death or survival in Cd^2+^-induced stress of renal PT cells in further detail. Considering the discrepancies *in vitro* (e.g., as in [100] *versus* [46]) and—more importantly—*in vivo* ([96] *versus* [46]), additional *in vitro* and *in vivo* studies with more compelling evidence are clearly required to prove or disprove the hypothesis that Cd^2+^ induces ACD in the kidney.

Although evidence suggests that ER stress, autophagy, and cell death coexist in Cd^2+^-treated PT cells *in vivo* and *in vitro* [41,96], the exact relationship between these different pathways, *i.e.*, the intracellular signals responsible for initiation (ROS? Ca^2+^? Or both?), and the sequence of activation of these cellular processes are unknown und need to be investigated in more detail.

In addition, because of the inconsistencies as well as possible limitations and fallacies associated with the use of renal PT cell lines of different origins, ideally autophagy should be investigated *in vivo*, for instance in tubular cells using GFP-LC3 reporter mice [115]. Cytosolic LC3-I is lipidated into LC3-II and recruited into autophagosomes, which can be identified as GFP-positive punctae in kidney sections of the GFP-LC3 mouse [115]. Hence, autophagy should be monitored in GFP-LC3 reporter mice during acute and chronic Cd^2+^ nephrotoxicity, according to protocols described elsewhere [116,117]. The protocols would be need to be optimized to monitor kinetics of autophagy induction and damage during Cd^2+^ stress in the PT of mice. With this information in mind, the role of autophagy on cell death or survival should then be investigated at time points where autophagy is prominent and/or damage is manifest in GFP-LC3 mice. The gold standard for determining the role of autophagy in Cd^2+^-induced nephrotoxicity is the generation of an autophagy knockout mouse. The autophagy-related protein ATG5 is crucial for autophagosome formation by conjugating with ATG12 to generate an E3 ubiquitin ligase-like enzyme required for autophagy [51]. To that end, tubular-specific Atg5-knockout mouse models (e.g., [94]) will be ideally suited to determine conclusively whether Cd^2+^ stress and toxicity triggers autophagy to elicit adaptive survival or cell death of renal PT cells.

Sphingolipids/ceramides are an important determinant of autophagy induction and execution and the role of sphingolipids/ceramides in relation to autophagy and Cd^2+^ nephrotoxicity is an issue that would need to be addressed in the future since Cd^2+^ causes alterations in the sphingolipid pool. Acid sphingomyelinase (ASMase) is associated with lysosomes in lysosomal (L-) ASMase, where it modulates autophagy [118,119]. L-ASMase is considered one of the major candidates for the production of ceramide in response to stress, including environmental insults [120]. Furthermore, stress induced by ceramides has been shown to cause cytoprotective autophagy or ACD depending on the ceramide chain length and/or activity of sphingolipid metabolizing enzymes (the so-called “ceramide autophagy paradox” [62,81]). Cd^2+^ interferes with both ceramide formation and L-ASMase in renal PT cells ([102] and unpublished data), which possibly underlies lysosomal dysfunction and block of lysosomal degradation. Consequently, it will be important to determine autophagy and cell fate in cultured PT cells exposed to Cd^2+^ where ASMase has been downregulated, or in Cd^2+^-exposed ASMase(^−/−^) mice [121].

Hopefully, a detailed understanding of the mechanisms of activation and regulation of autophagy and its role in cell fate decisions may ultimately contribute to the development of novel strategies for prevention and therapy of acute and chronic Cd^2+^ nephrotoxicity.

## References

[1] Nawrot T.S., Staessen J.A., Roels H.A., Munters E., Cuypers A., Richart T., Ruttens A., Smeets K., Clijsters H., Vangronsveld J. (2010). Cadmium exposure in the population: From health risks to strategies of prevention. Biometals.

[2] Satarug S., Moore M.R. (2004). Adverse health effects of chronic exposure to low-level cadmium in foodstuffs and cigarette smoke. Environ. Health Perspect..

[3] (2011). CONTAM Statement on tolerable weekly intake for cadmium. EFSA J..

[4] Jarup L., Akesson A. (2009). Current status of cadmium as an environmental health problem. Toxicol. Appl. Pharmacol..

[5] Thévenod F. (2003). Nephrotoxicity and the proximal tubule. Insights from cadmium. Nephron Physiol..

[6] Thévenod F. (2009). Cadmium and cellular signaling cascades: To be or not to be?. Toxicol. Appl. Pharmacol..

[7] Thévenod F., Lee W.K. (2013). Cadmium and cellular signaling cascades: Interactions between cell death and survival pathways. Arch. Toxicol..

[8] Thévenod F., Lee W.K. (2013). Toxicology of cadmium and its damage to Mammalian organs. Met. Ions Life Sci..

[9] Ferraro P.M., Costanzi S., Naticchia A., Sturniolo A., Gambaro G. (2010). Low level exposure to cadmium increases the risk of chronic kidney disease: Analysis of the NHANES 1999–2006. BMC Public Health.

[10] Hartwig A. (2013). Cadmium and cancer. Met. Ions Life Sci..

[11] Waisberg M., Joseph P., Hale B., Beyersmann D. (2003). Molecular and cellular mechanisms of cadmium carcinogenesis. Toxicology.

[12] Hanahan D., Weinberg R.A. (2011). Hallmarks of cancer: The next generation. Cell.

[13] Galluzzi L., Bravo-San Pedro J.M., Vitale I., Aaronson S.A., Abrams J.M., Adam D., Alnemri E.S., Altucci L., Andrews D., Annicchiarico-Petruzzelli M. (2015). Essential *versus* accessory aspects of cell death: Recommendations of the NCCD 2015. Cell Death Differ..

[14] Galluzzi L., Vitale I., Abrams J.M., Alnemri E.S., Baehrecke E.H., Blagosklonny M.V., Dawson T.M., Dawson V.L., El-Deiry W.S., Fulda S. (2012). Molecular definitions of cell death subroutines: Recommendations of the Nomenclature Committee on Cell Death 2012. Cell Death Differ..

[15] Green D.R., Galluzzi L., Kroemer G. (2014). Cell biology. Metabolic control of cell death. Science.

[16] Mizushima N. (2007). Autophagy: Process and function. Genes Dev..

[17] Mizushima N., Komatsu M. (2011). Autophagy: Renovation of cells and tissues. Cell.

[18] Kroemer G., Marino G., Levine B. (2010). Autophagy and the integrated stress response. Mol. Cell.

[19] Yang Z., Klionsky D.J. (2010). Mammalian autophagy: Core molecular machinery and signaling regulation. Curr. Opin. Cell Biol..

[20] Li L., Ishdorj G., Gibson S.B. (2012). Reactive oxygen species regulation of autophagy in cancer: Implications for cancer treatment. Free Radic. Biol. Med..

[21] Scherz-Shouval R., Elazar Z. (2011). Regulation of autophagy by ROS: Physiology and pathology. Trends Biochem. Sci..

[22] Decuypere J.P., Bultynck G., Parys J.B. (2011). A dual role for Ca^2+^ in autophagy regulation. Cell Calcium.

[23] Chen Y., McMillan-Ward E., Kong J., Israels S.J., Gibson S.B. (2007). Mitochondrial electron-transport-chain inhibitors of complexes I and II induce autophagic cell death mediated by reactive oxygen species. J. Cell Sci..

[24] Yoon S., Woo S.U., Kang J.H., Kim K., Kwon M.H., Park S., Shin H.J., Gwak H.S., Chwae Y.J. (2010). STAT3 transcriptional factor activated by reactive oxygen species induces IL6 in starvation-induced autophagy of cancer cells. Autophagy.

[25] Bellot G., Garcia-Medina R., Gounon P., Chiche J., Roux D., Pouyssegur J., Mazure N.M. (2009). Hypoxia-induced autophagy is mediated through hypoxia-inducible factor induction of BNIP3 and BNIP3L via their BH3 domains. Mol. Cell. Biol..

[26] Burton T.R., Gibson S.B. (2009). The role of Bcl-2 family member BNIP3 in cell death and disease: NIPping at the heels of cell death. Cell Death Differ..

[27] Cardenas C., Foskett J.K. (2012). Mitochondrial Ca(2+) signals in autophagy. Cell Calcium.

[28] Decuypere J.P., Welkenhuyzen K., Luyten T., Ponsaerts R., Dewaele M., Molgo J., Agostinis P., Missiaen L., de Smedt H., Parys J.B. (2011). Ins(1,4,5)P3 receptor-mediated Ca2+ signaling and autophagy induction are interrelated. Autophagy.

[29] Lam D., Kosta A., Luciani M.F., Golstein P. (2008). The inositol 1,4,5-trisphosphate receptor is required to signal autophagic cell death. Mol. Biol. Cell.

[30] Sakaki K., Wu J., Kaufman R.J. (2008). Protein kinase Ctheta is required for autophagy in response to stress in the endoplasmic reticulum. J. Biol. Chem..

[31] Hoyer-Hansen M., Jaattela M. (2007). Connecting endoplasmic reticulum stress to autophagy by unfolded protein response and calcium. Cell Death Differ..

[32] Ron D., Walter P. (2007). Signal integration in the endoplasmic reticulum unfolded protein response. Nat. Rev. Mol. Cell Biol..

[33] Woehlbier U., Hetz C. (2011). Modulating stress responses by the UPRosome: A matter of life and death. Trends Biochem. Sci..

[34] Senft D., Ronai Z.A. (2015). UPR, autophagy, and mitochondria crosstalk underlies the ER stress response. Trends Biochem. Sci..

[35] McCullough K.D., Martindale J.L., Klotz L.O., Aw T.Y., Holbrook N.J. (2001). Gadd153 sensitizes cells to endoplasmic reticulum stress by down-regulating Bcl2 and perturbing the cellular redox state. Mol. Cell. Biol..

[36] Schroder M., Kaufman R.J. (2005). The mammalian unfolded protein response. Annu. Rev. Biochem..

[37] Tabas I., Ron D. (2011). Integrating the mechanisms of apoptosis induced by endoplasmic reticulum stress. Nat. Cell Biol..

[38] Hiramatsu N., Kasai A., Du S., Takeda M., Hayakawa K., Okamura M., Yao J., Kitamura M. (2007). Rapid, transient induction of ER stress in the liver and kidney after acute exposure to heavy metal: Evidence from transgenic sensor mice. FEBS Lett..

[39] Yokouchi M., Hiramatsu N., Hayakawa K., Okamura M., Du S., Kasai A., Takano Y., Shitamura A., Shimada T., Yao J. (2008). Involvement of selective reactive oxygen species upstream of proapoptotic branches of unfolded protein response. J. Biol. Chem..

[40] Biagioli M., Pifferi S., Ragghianti M., Bucci S., Rizzuto R., Pinton P. (2008). Endoplasmic reticulum stress and alteration in calcium homeostasis are involved in cadmium-induced apoptosis. Cell Calcium.

[41] Lee W.K., Chakraborty P.K., Roussa E., Wolff N.A., Thévenod F. (2012). ERK1/2-dependent bestrophin-3 expression prevents ER-stress-induced cell death in renal epithelial cells by reducing CHOP. Biochim. Biophys. Acta.

[42] Permenter M.G., Lewis J.A., Jackson D.A. (2011). Exposure to nickel, chromium, or cadmium causes distinct changes in the gene expression patterns of a rat liver derived cell line. PLoS One.

[43] Yokouchi M., Hiramatsu N., Hayakawa K., Kasai A., Takano Y., Yao J., Kitamura M. (2007). Atypical, bidirectional regulation of cadmium-induced apoptosis via distinct signaling of unfolded protein response. Cell Death Differ..

[44] Chakraborty P.K., Lee W.K., Molitor M., Wolff N.A., Thévenod F. (2010). Cadmium induces Wnt signaling to upregulate proliferation and survival genes in sub-confluent kidney proximal tubule cells. Mol. Cancer.

[45] Komoike Y., Inamura H., Matsuoka M. (2012). Effects of salubrinal on cadmium-induced apoptosis in HK-2 human renal proximal tubular cells. Arch. Toxicol..

[46] Kato H., Katoh R., Kitamura M. (2013). Dual regulation of cadmium-induced apoptosis by mTORC1 through selective induction of IRE1 branches in unfolded protein response. PLoS One.

[47] Ji Y.L., Wang H., Zhao X.F., Wang Q., Zhang C., Zhang Y., Zhao M., Chen Y.H., Meng X.H., Xu D.X. (2011). Crosstalk between endoplasmic reticulum stress and mitochondrial pathway mediates cadmium-induced germ cell apoptosis in testes. Toxicol. Sci..

[48] Ogata M., Hino S., Saito A., Morikawa K., Kondo S., Kanemoto S., Murakami T., Taniguchi M., Tanii I., Yoshinaga K. (2006). Autophagy is activated for cell survival after endoplasmic reticulum stress. Mol. Cell. Biol..

[49] Ding W.X., Ni H.M., Gao W., Hou Y.F., Melan M.A., Chen X., Stolz D.B., Shao Z.M., Yin X.M. (2007). Differential effects of endoplasmic reticulum stress-induced autophagy on cell survival. J. Biol. Chem..

[50] Ullman E., Fan Y., Stawowczyk M., Chen H.M., Yue Z., Zong W.X. (2008). Autophagy promotes necrosis in apoptosis-deficient cells in response to ER stress. Cell Death Differ..

[51] Marino G., Niso-Santano M., Baehrecke E.H., Kroemer G. (2014). Self-consumption: The interplay of autophagy and apoptosis. Nat. Rev. Mol. Cell Biol..

[52] Shen S., Kepp O., Kroemer G. (2012). The end of autophagic cell death?. Autophagy.

[53] Kroemer G., Levine B. (2008). Autophagic cell death: The story of a misnomer. Nat. Rev. Mol. Cell Biol..

[54] Djavaheri-Mergny M., Maiuri M.C., Kroemer G. (2010). Cross talk between apoptosis and autophagy by caspase-mediated cleavage of Beclin 1. Oncogene.

[55] Betin V.M., Lane J.D. (2009). Caspase cleavage of Atg4D stimulates GABARAP-L1 processing and triggers mitochondrial targeting and apoptosis. J. Cell Sci..

[56] Yousefi S., Perozzo R., Schmid I., Ziemiecki A., Schaffner T., Scapozza L., Brunner T., Simon H.U. (2006). Calpain-mediated cleavage of Atg5 switches autophagy to apoptosis. Nat. Cell Biol..

[57] Madden D.T., Egger L., Bredesen D.E. (2007). A calpain-like protease inhibits autophagic cell death. Autophagy.

[58] Liu Y., Shoji-Kawata S., Sumpter R.M., Wei Y., Ginet V., Zhang L., Posner B., Tran K.A., Green D.R., Xavier R.J. (2013). Autosis is a Na^+^,K^+^-ATPase-regulated form of cell death triggered by autophagy-inducing peptides, starvation, and hypoxia-ischemia. Proc. Natl. Acad. Sci. USA.

[59] Clarke P.G., Puyal J. (2012). Autophagic cell death exists. Autophagy.

[60] Chen Y., Klionsky D.J. (2011). The regulation of autophagy—Unanswered questions. J. Cell Sci..

[61] Denton D., Nicolson S., Kumar S. (2012). Cell death by autophagy: Facts and apparent artefacts. Cell Death Differ..

[62] Dany M., Ogretmen B. (2015). Ceramide induced mitophagy and tumor suppression. Biochim. Biophys. Acta.

[63] Nelson C., Baehrecke E.H. (2014). Eaten to death. FEBS J..

[64] Fulda S., Kogel D. (2015). Cell death by autophagy: Emerging molecular mechanisms and implications for cancer therapy. Oncogene.

[65] Denton D., Xu T., Kumar S. (2015). Autophagy as a pro-death pathway. Immunol. Cell Biol..

[66] Liu Y., Levine B. (2015). Autosis and autophagic cell death: The dark side of autophagy. Cell Death Differ..

[67] Shimizu S., Konishi A., Nishida Y., Mizuta T., Nishina H., Yamamoto A., Tsujimoto Y. (2010). Involvement of JNK in the regulation of autophagic cell death. Oncogene.

[68] Shimizu S., Kanaseki T., Mizushima N., Mizuta T., Arakawa-Kobayashi S., Thompson C.B., Tsujimoto Y. (2004). Role of Bcl-2 family proteins in a non-apoptotic programmed cell death dependent on autophagy genes. Nat. Cell Biol..

[69] Yu L., Alva A., Su H., Dutt P., Freundt E., Welsh S., Baehrecke E.H., Lenardo M.J. (2004). Regulation of an ATG7-beclin 1 program of autophagic cell death by caspase-8. Science.

[70] Yu L., Wan F., Dutta S., Welsh S., Liu Z., Freundt E., Baehrecke E.H., Lenardo M. (2006). Autophagic programmed cell death by selective catalase degradation. Proc. Natl. Acad. Sci. USA.

[71] Pattingre S., Tassa A., Qu X., Garuti R., Liang X.H., Mizushima N., Packer M., Schneider M.D., Levine B. (2005). Bcl-2 antiapoptotic proteins inhibit Beclin 1-dependent autophagy. Cell.

[72] Lamy L., Ngo V.N., Emre N.C., Shaffer A.L., Yang Y., Tian E., Nair V., Kruhlak M.J., Zingone A., Landgren O. (2013). Control of autophagic cell death by caspase-10 in multiple myeloma. Cancer Cell.

[73] Reef S., Zalckvar E., Shifman O., Bialik S., Sabanay H., Oren M., Kimchi A. (2006). A short mitochondrial form of p19ARF induces autophagy and caspase-independent cell death. Mol. Cell.

[74] Elgendy M., Sheridan C., Brumatti G., Martin S.J. (2011). Oncogenic Ras-induced expression of Noxa and Beclin-1 promotes autophagic cell death and limits clonogenic survival. Mol. Cell.

[75] Pelled D., Raveh T., Riebeling C., Fridkin M., Berissi H., Futerman A.H., Kimchi A. (2002). Death-associated protein (DAP) kinase plays a central role in ceramide-induced apoptosis in cultured hippocampal neurons. J. Biol. Chem..

[76] Widau R.C., Jin Y., Dixon S.A., Wadzinski B.E., Gallagher P.J. (2010). Protein phosphatase 2A (PP2A) holoenzymes regulate death-associated protein kinase (DAPK) in ceramide-induced anoikis. J. Biol. Chem..

[77] Bialik S., Kimchi A. (2010). Lethal weapons: DAP-kinase, autophagy and cell death: DAP-kinase regulates autophagy. Curr. Opin. Cell Biol..

[78] Yukawa K., Shirasawa N., Ohshima A., Mune M., Kimura A., Bai T., Tsubota Y., Owada-Makabe K., Tanaka T., Kishino M. (2004). Death-associated protein kinase localization to human renal tubule cells, and increased expression of chronic obstructive uropathy in rats. J. Nephrol..

[79] Kishino M., Yukawa K., Hoshino K., Kimura A., Shirasawa N., Otani H., Tanaka T., Owada-Makabe K., Tsubota Y., Maeda M. (2004). Deletion of the kinase domain in death-associated protein kinase attenuates tubular cell apoptosis in renal ischemia-reperfusion injury. J. Am. Soc. Nephrol..

[80] Gozuacik D., Bialik S., Raveh T., Mitou G., Shohat G., Sabanay H., Mizushima N., Yoshimori T., Kimchi A. (2008). DAP-kinase is a mediator of endoplasmic reticulum stress-induced caspase activation and autophagic cell death. Cell Death Differ..

[81] Jiang W., Ogretmen B. (2014). Autophagy paradox and ceramide. Biochim. Biophys. Acta.

[82] Sentelle R.D., Senkal C.E., Jiang W., Ponnusamy S., Gencer S., Selvam S.P., Ramshesh V.K., Peterson Y.K., Lemasters J.J., Szulc Z.M. (2012). Ceramide targets autophagosomes to mitochondria and induces lethal mitophagy. Nat. Chem. Biol..

[83] Maskey D., Yousefi S., Schmid I., Zlobec I., Perren A., Friis R., Simon H.U. (2013). ATG5 is induced by DNA-damaging agents and promotes mitotic catastrophe independent of autophagy. Nat. Commun..

[84] Surova O., Zhivotovsky B. (2013). Various modes of cell death induced by DNA damage. Oncogene.

[85] Chiarelli R., Agnello M., Bosco L., Roccheri M.C. (2014). Sea urchin embryos exposed to cadmium as an experimental model for studying the relationship between autophagy and apoptosis. Mar. Environ. Res..

[86] Pi H., Xu S., Zhang L., Guo P., Li Y., Xie J., Tian L., He M., Lu Y., Li M. (2013). Dynamin 1-like-dependent mitochondrial fission initiates overactive mitophagy in the hepatotoxicity of cadmium. Autophagy.

[87] Son Y.O., Pratheeshkumar P., Roy R.V., Hitron J.A., Wang L., Zhang Z., Shi X. (2014). Nrf2/p62 signaling in apoptosis resistance and its role in cadmium-induced carcinogenesis. J. Biol. Chem..

[88] Lieberthal W., Levine J.S. (2012). Mammalian target of rapamycin and the kidney. II. Pathophysiology and therapeutic implications. Am. J. Physiol. Renal Physiol..

[89] Huber T.B., Edelstein C.L., Hartleben B., Inoki K., Dong Z., Koya D., Kume S., Lieberthal W., Pallet N., Quiroga A. (2012). Emerging role of autophagy in kidney function, diseases and aging. Autophagy.

[90] Fougeray S., Pallet N. (2015). Mechanisms and biological functions of autophagy in diseased and ageing kidneys. Nat. Rev. Nephrol..

[91] Sureshbabu A., Ryter S.W., Choi M.E. (2015). Oxidative stress and autophagy: Crucial modulators of kidney injury. Redox Biol..

[92] Rewa O., Bagshaw S.M. (2014). Acute kidney injury-epidemiology, outcomes and economics. Nat. Rev. Nephrol..

[93] Kimura T., Takabatake Y., Takahashi A., Kaimori J.Y., Matsui I., Namba T., Kitamura H., Niimura F., Matsusaka T., Soga T. (2011). Autophagy protects the proximal tubule from degeneration and acute ischemic injury. J. Am. Soc. Nephrol..

[94] Liu S., Hartleben B., Kretz O., Wiech T., Igarashi P., Mizushima N., Walz G., Huber T.B. (2012). Autophagy plays a critical role in kidney tubule maintenance, aging and ischemia-reperfusion injury. Autophagy.

[95] Wang S.H., Shih Y.L., Ko W.C., Wei Y.H., Shih C.M. (2008). Cadmium-induced autophagy and apoptosis are mediated by a calcium signaling pathway. Cell. Mol. Life Sci..

[96] Chargui A., Zekri S., Jacquillet G., Rubera I., Ilie M., Belaid A., Duranton C., Tauc M., Hofman P., Poujeol P. (2011). Cadmium-induced autophagy in rat kidney: An early biomarker of subtoxic exposure. Toxicol. Sci..

[97] Wei X., Qi Y., Zhang X., Qiu Q., Gu X., Tao C., Huang D., Zhang Y. (2014). Cadmium induces mitophagy through ROS-mediated PINK1/Parkin pathway. Toxicol. Mech. Methods.

[98] Fujiki K., Inamura H., Matsuoka M. (2014). PI3K signaling mediates diverse regulation of ATF4 expression for the survival of HK-2 cells exposed to cadmium. Arch. Toxicol..

[99] Li J., Kim S.G., Blenis J. (2014). Rapamycin: One drug, many effects. Cell Metab..

[100] Thévenod F., Lee W.K., Wolff N.A. (2015). Rapamycin: A therapy of choice for endoplasmic reticulum stress-induced renal proximal tubule toxicity?. Toxicology.

[101] Lee W.K., Abouhamed M., Thévenod F. (2006). Caspase-dependent and -independent pathways for cadmium-induced apoptosis in cultured kidney proximal tubule cells. Am. J. Physiol. Renal Physiol..

[102] Lee W.K., Torchalski B., Thévenod F. (2007). Cadmium-induced ceramide formation triggers calpain-dependent apoptosis in cultured kidney proximal tubule cells. Am. J. Physiol. Cell Physiol..

[103] Lee W.K., Bork U., Gholamrezaei F., Thévenod F. (2005). Cd2+-induced cytochrome c release in apoptotic proximal tubule cells: Role of mitochondrial permeability transition pore and Ca^2+^ uniporter. Am. J. Physiol. Renal Physiol..

[104] Thévenod F., Friedmann J.M. (1999). Cadmium-mediated oxidative stress in kidney proximal tubule cells induces degradation of Na^+^/K^+^-ATPase through proteasomal and endo-/lysosomal proteolytic pathways. FASEB J..

[105] Xu C., Kim N.G., Gumbiner B.M. (2009). Regulation of protein stability by GSK3 mediated phosphorylation. Cell Cycle.

[106] Yang J., Takahashi Y., Cheng E., Liu J., Terranova P.F., Zhao B., Thrasher J.B., Wang H.G., Li B. (2010). GSK-3beta promotes cell survival by modulating Bif-1-dependent autophagy and cell death. J. Cell Sci..

[107] Yang L.Y., Wu K.H., Chiu W.T., Wang S.H., Shih C.M. (2009). The cadmium-induced death of mesangial cells results in nephrotoxicity. Autophagy.

[108] Lee W.K., Thévenod F. (2008). Novel roles for ceramides, calpains and caspases in kidney proximal tubule cell apoptosis: Lessons from *in vitro* cadmium toxicity studies. Biochem. Pharmacol..

[109] Lieberthal W., Levine J.S. (2012). Mammalian target of rapamycin and the kidney. I. The signaling pathway. Am. J. Physiol. Renal Physiol..

[110] Li J., Xu Z., Jiang L., Mao J., Zeng Z., Fang L., He W., Yuan W., Yang J., Dai C. (2014). Rictor/mTORC2 protects against cisplatin-induced tubular cell death and acute kidney injury. Kidney Int..

[111] Sarbassov D.D., Ali S.M., Sengupta S., Sheen J.H., Hsu P.P., Bagley A.F., Markhard A.L., Sabatini D.M. (2006). Prolonged rapamycin treatment inhibits mTORC2 assembly and Akt/PKB. Mol. Cell.

[112] Smith K.D., Wrenshall L.E., Nicosia R.F., Pichler R., Marsh C.L., Alpers C.E., Polissar N., Davis C.L. (2003). Delayed graft function and cast nephropathy associated with tacrolimus plus rapamycin use. J. Am. Soc. Nephrol..

[113] Klionsky D.J., Abdalla F.C., Abeliovich H., Abraham R.T., Acevedo-Arozena A., Adeli K., Agholme L., Agnello M., Agostinis P., Aguirre-Ghiso J.A. (2012). Guidelines for the use and interpretation of assays for monitoring autophagy. Autophagy.

[114] Messner B., Ploner C., Laufer G., Bernhard D. (2012). Cadmium activates a programmed, lysosomal membrane permeabilization-dependent necrosis pathway. Toxicol. Lett..

[115] Mizushima N., Yamamoto A., Matsui M., Yoshimori T., Ohsumi Y. (2004). In vivo analysis of autophagy in response to nutrient starvation using transgenic mice expressing a fluorescent autophagosome marker. Mol. Biol. Cell.

[116] Liu Y.P., Liu J., Palmiter R.D., Klaassen C.D. (1996). Metallothionein-I-transgenic mice are not protected from acute cadmium-metallothionein-induced nephrotoxicity. Toxicol. Appl. Pharmacol..

[117] Thijssen S., Maringwa J., Faes C., Lambrichts I., van Kerkhove E. (2007). Chronic exposure of mice to environmentally relevant, low doses of cadmium leads to early renal damage, not predicted by blood or urine cadmium levels. Toxicology.

[118] Gabande-Rodriguez E., Boya P., Labrador V., Dotti C.G., Ledesma M.D. (2014). High sphingomyelin levels induce lysosomal damage and autophagy dysfunction in Niemann Pick disease type A. Cell Death Differ..

[119] Perrotta C., Cervia D., De Palma C., Assi E., Pellegrino P., Bassi M.T., Clementi E. (2015). The emerging role of Acid Sphingomyelinase in autophagy. Apoptosis.

[120] Jenkins R.W., Canals D., Hannun Y.A. (2009). Roles and regulation of secretory and lysosomal acid sphingomyelinase. Cell Signal..

[121] Hua G., Kolesnick R. (2013). Using ASMase knockout mice to model human diseases. Handb. Exp. Pharmacol..

